# *Pseudomonas aeruginosa mgtC* gene is under the control of PhoP and CbrAB regulators, and its expression can be visualized in macrophages

**DOI:** 10.1128/spectrum.00064-26

**Published:** 2026-08-05

**Authors:** Anne Bonhoure, Pauline Nogaret, Valérie Perez, Flore Nilly, Sylvaine Huc-Brandt, Malika Moussouni, Robert E. W. Hancock, Virginie Molle, Anne-Béatrice Blanc-Potard

**Affiliations:** 1 Laboratory of Pathogens and Host Immunity (LPHI), Université de Montpellier, CNRS 27037, Montpellier, France; 2 Centre for Microbial Diseases and Immunity Research, University of British Columbia 8166, Vancouver, British Columbia, Canada; University of Guelph College of Biological Science, Guelph, Ontario, Canada

**Keywords:** *Pseudomonas aeruginosa*, MgtC, macrophages, two-component system, zebrafish

## Abstract

The MgtC virulence factor is important during the intramacrophage stage in both classical intracellular pathogens, such as *Salmonella* Typhimurium, and in extracellular bacteria that transiently encounter intracellular environments during infection, such as *Pseudomonas aeruginosa*. In these different pathogens, *mgtC* expression is induced *in vitro* by magnesium ion depletion, a condition reported to mimic the macrophage environment. Here, we developed an unstable GFP reporter system to monitor in real time the transcriptional activation of the *P. aeruginosa mgtC* promoter. After *in vitro* validation in magnesium-defined media, this reporter system allowed visualization of the *mgtC* promoter induction in a subset of bacteria when *P. aeruginosa* localized inside cultured macrophages. In addition, although rare under our experimental conditions, *in vivo* activation of the *mgtC* promoter was observed for the first time within macrophages of live, infected zebrafish larvae, a cutting-edge vertebrate model for real-time imaging. While MgtC regulation in *Salmonella* is mediated by the magnesium-responsive PhoPQ two-component system, its regulation in *P. aeruginosa* remained unknown. The use of mutant strains for two-component regulatory systems revealed that the PhoP regulator, but not by its cognate sensor PhoQ, was required to activate *P. aeruginosa* MgtC expression *in vitro*. Unexpectedly, CbrAB, a two-component system specific to *Pseudomonas* species, was also involved in *P. aeruginosa* MgtC regulation. Both PhoP and CbrB regulatory proteins were found to directly bind the *mgtC* promoter, supporting a dual transcriptional control. These findings reveal substantial differences in *mgtC* gene regulation in different bacterial pathogens, reflecting distinct strategies to drive appropriate expression of a shared virulence factor involved in macrophage adaptation.

**IMPORTANCE:**

The adaptation of bacterial pathogens to the host intracellular microenvironment requires tight and rapid regulation of specific genes, and investigating the *in vivo* transcriptional dynamics of such genes is a major challenge. Here, we focused on the expression of *mgtC*, a gene important for adaptation to the intramacrophage environment in classical intracellular pathogens, such as *Salmonella* Typhimurium, and bacteria with a transient intracellular lifestyle, such as *Pseudomonas aeruginosa*. An unstable GFP reporter system was designed to monitor the transcriptional dynamics of *P. aeruginosa mgtC*. The use of this reporter system in a state-of-the-art vertebrate model for live imaging, the zebrafish embryo, allowed *in vivo* tracking of *P. aeruginosa mgtC* promoter activation inside macrophages in a living host. Furthermore, the expression of *P. aeruginosa mgtC* was found to be regulated through a mechanism distinct from that of *Salmonella* MgtC, since it involves the PhoP regulatory protein, but not the PhoQ sensor, and the *Pseudomonas*-specific CbrAB two-component system, reflecting diverse, finely tuned strategies to control a virulence factor shared by several major human pathogens.

## INTRODUCTION

*Pseudomonas aeruginosa* is a Gram-negative environmental bacterium able to thrive in very diverse habitats, due to its metabolic versatility and the presence of a large number of regulators, including two-component systems (1). *P. aeruginosa* is also an opportunistic human pathogen, responsible for acute and chronic infections. Despite being described as an extracellular pathogen, increasing evidence indicates that *P. aeruginosa* can reside, at least transiently, inside cultured epithelial cells and macrophages (2). During infection, *P. aeruginosa* has been reported to be engulfed by alveolar macrophages in the lungs of mice shortly after the onset of pulmonary infections (3), and intracellular *P. aeruginosa* have recently been observed in the airway epithelia of human cystic fibrosis (CF) lung explants (4). To track intracellular *P. aeruginosa in vivo*, the zebrafish embryo has emerged as a state-of-the-art infection model, which allows precise real-time imaging (5). In this model, *P. aeruginosa* has been observed inside macrophages during both acute (6–8) and persistent infections (9, 10).

The expression of specialized bacterial virulence factors is crucial for adaptation to the host intracellular environment, particularly the stressful conditions encountered within macrophages. Among these, the bacterial MgtC protein provides a striking example of a virulence factor that promotes intramacrophage adaptation in both intracellular pathogens, such as *Salmonella enterica* serovar Typhimurium, and extracellular pathogens, such as *P. aeruginosa* (11, 12). MgtC is a membrane protein that modulates the intracellular level of phosphate and ATP in bacteria (13, 14). In line with its role within cultured macrophages, MgtC is expressed inside macrophages (11, 12, 15, 16) and contributes to successful infection in animal models in a macrophage-dependent manner (11, 14). MgtC is thus considered a marker of bacterial pathogens adapted to an intramacrophage stage (17).

In addition to being expressed in the macrophage environment, *mgtC* expression is induced in both *S*. Typhimurium and *P. aeruginosa* under conditions of Mg^2+^ limitation (11, 18). The regulation of *mgtC* has been primarily studied in *S*. Typhimurium (15), where Mg^2+^-dependent expression is mediated by the PhoPQ two-component system (19), a key regulator of *Salmonella* virulence. *P. aeruginosa* harbors an analogous PhoPQ system, although distinct from that of *Salmonella* (20, 21), which contributes to virulence and resistance to antimicrobial peptides (22, 23). However, transcriptomic analysis using *phoP* or *phoQ* mutants, as well as chromatin immunoprecipitation sequencing approaches, failed to clearly implicate *P. aeruginosa* PhoPQ in the regulation of *mgtC* (20, 22, 24).

In the present study, we investigated the dynamics and regulation of *P. aeruginosa mgtC* expression in various experimental contexts. To this end, we developed a fluorescence-based approach using an unstable Green Fluorescent Protein (GFP) reporter system, enabling real-time monitoring of *mgtC* promoter induction both in bacterial cultures and during host infection. A transient induction of the *mgtC* promoter was observed in a subset of bacteria upon phagocytosis by cultured macrophages, while *mgtC* promoter-driven GFP expression was also observed *in vivo* within macrophages of infected zebrafish larvae. Investigating the regulation of *mgtC* by two-component regulatory systems revealed that the activation of MgtC expression was dependent on the PhoP regulator, but not its cognate sensor PhoQ, and on CbrAB, a two-component system specific to *Pseudomonas* species.

## RESULTS

### *In vitro* validation of a reporter system expressing unstable GFP under the control of the *mgtC* promoter

**Fig 1:**
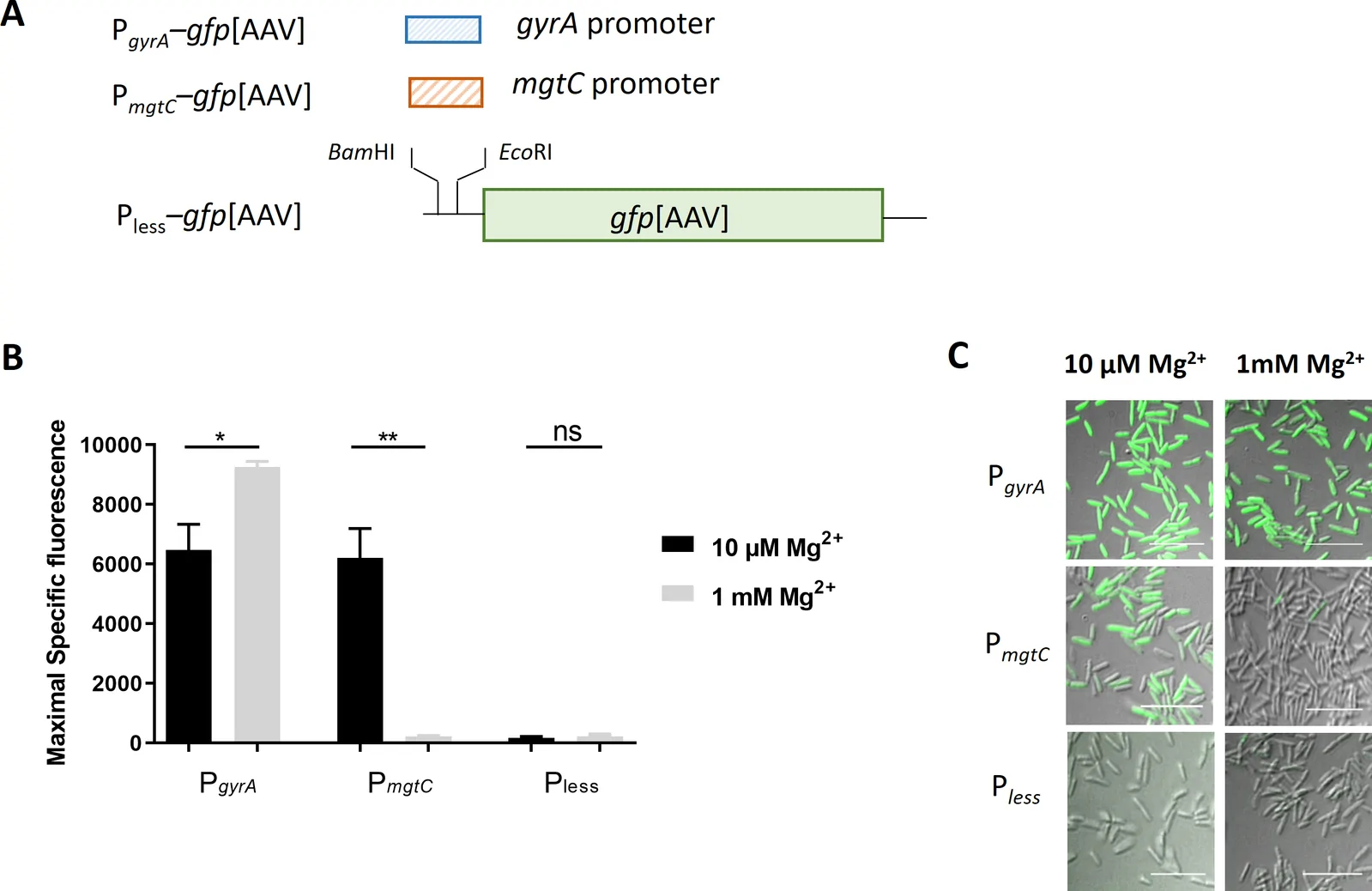
*In vitro* validation of a reporter system for *P. aeruginosa mgtC* promoter expression using unstable GFP. (**A**) Schematic representation of the constructs used in the study. In addition to the *mgtC* promoter, the *gyrA* promoter was included as a strong constitutive promoter, and a promoterless plasmid was used as a negative control. (**B**) Measurement of specific fluorescence following a shift of bacterial cultures to NCE medium containing 10 µM or 1 mM Mg^2+^. Specific fluorescence is expressed as the ratio of GFP fluorescence to absorbance at 600 nm. The maximal specific fluorescence intensity is reported (this maximal value is obtained approximately 2 h after the shift to NCE medium). Data represent the mean ± standard error from three to five independent experiments. Statistical analysis was performed using an unpaired *t*-test (*P < 0.05, **P < 0.01; ns, not significant). (**C**) Visualization of bacteria by fluorescence microscopy. The bacteria were grown for 2 h in the presence of 10 µM or 1 mM Mg^2+^. Merged phase-contrast and epifluorescence images are shown. The scale bars represent 10 μm.

A fluorescent construct was developed to allow real-time visualization of *P. aeruginosa mgtC* promoter activation. The promoter sequence of the *mgtC* gene was inserted into the vector pPROBE*–gfp*[AAV] to generate a transcriptional fusion with the reporter gene *gfp*[AAV], which encodes an unstable GFP due to an AAV degradation tag at its C-terminus, validated in both *Escherichia coli* and *Pseudomonas* species (25) (Fig. 1A). As a positive control, the constitutive *gyrA* promoter, previously shown to be similarly expressed in low-magnesium medium, high-magnesium medium, and macrophage environment (11), was cloned into the same vector (Fig. 1A). A promoterless plasmid was used as a negative control. All constructs were introduced into the wild-type *P. aeruginosa* strain PAO1.

To validate the reporter system, fluorescence was measured *in vitro* in bacterial cultures containing either 10 µM Mg^2+^ or 1 mM Mg^2+^, conditions known to respectively induce or repress *mgtC* expression based on RT-qPCR analyses (11). As expected, fluorescence from the P*_mgtC_–gfp*[AAV] strain was strongly induced in 10 µM Mg^2+^ when compared to 1 mM Mg^2+^, where fluorescence remained at background levels, similar to the negative control (Fig. 1B). At maximum fluorescence intensity, the signal for the *mgtC* promoter in low Mg^2+^ was 50-fold higher than in high Mg^2+^ (Fig. 1B). Conversely, the strain harboring the control P*_gyrA_–gfp*[AAV] fusion showed high fluorescence levels in both conditions, with no increased expression in low Mg^2+^ (in contrast, a ~25% reduction is observed) (Fig. 1B).

We next visualized bacteria harboring the *gfp*[AAV] plasmids by fluorescence microscopy after growth in 10 µM or 1 mM Mg^2+^. P*_mgtC_–gfp*[AAV] bacteria showed clear fluorescence, but with heterogeneous expression between bacteria, ranging from very strong to not observable, under inducing conditions (10 µM Mg^2+^), while no fluorescence, except for extremely rare bacteria, was observed in 1 mM Mg^2+^ (Fig. 1C). In contrast, the positive control (P*_gyrA_–gfp*[AAV]) displayed homogeneous fluorescence in both conditions, and no fluorescence was visualized with the negative control (P_less_*–gfp*[AAV]).

We also assessed the level of chromosomally encoded MgtC and plasmid-encoded GFP[AAV] proteins, both under the control of the *mgtC* promoter, in a single strain. Total lysates were prepared at different time points from PAO1 bacteria harboring P*_mgtC_–gfp*[AAV] grown for 3 h in inducing conditions (10 µM Mg^2+^) and shifted to repressing conditions (1 mM Mg^2+^). Western blotting was carried out with anti-MgtC and anti-GFP antibodies. No MgtC nor GFP[AAV] signal was detected at early time points, indicating a strong repression of the *mgtC* promoter. Both MgtC and GFP[AAV] proteins were detected after 2 h in 10 µM Mg^2+^. As anticipated, the level of GFP[AAV] decreased rapidly after the shift to 1 mM Mg^2+^, whereas MgtC protein was more stable over time (Fig. S1 available at https://doi.org/10.5281/zenodo.20323693)

Altogether, these results demonstrate that we successfully constructed a functional dynamic reporter system to monitor induction of the *P. aeruginosa mgtC* promoter.

### Visualization of *mgtC* promoter activity inside macrophages using a cell line or an *in vivo* infection model

**Fig 2:**
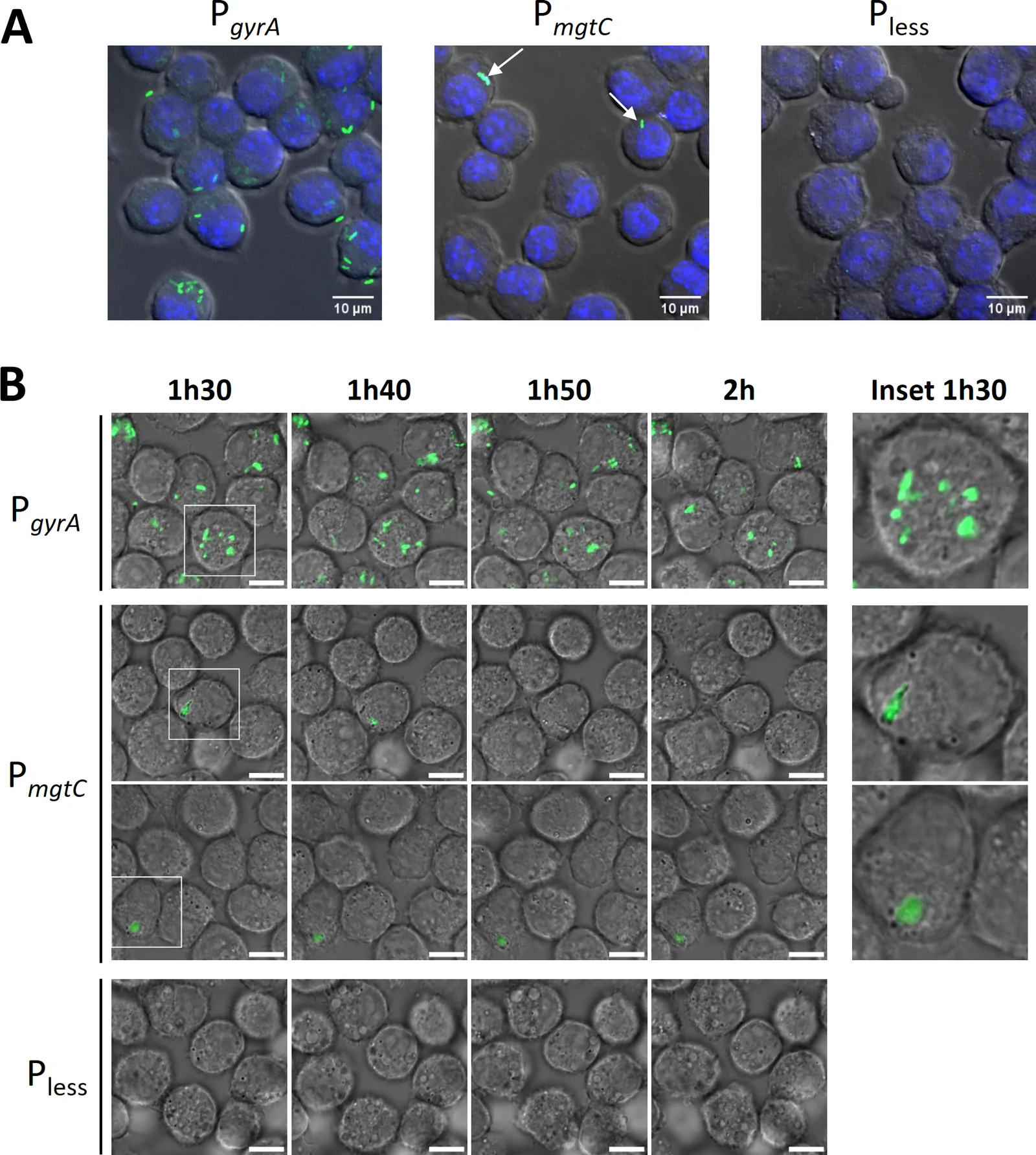
Visualization of *P. aeruginosa mgtC* promoter induction inside cultured macrophages. *P. aeruginosa* PAO1 strains carrying the reporter gene *gfp*[AAV] under *gyrA* promoter (P*_gyrA_*), *mgtC* promoter (P*_mgtC_*), or without promoter (P_less_) were used to infect J774 macrophages. (**A**) Two hours after infection, macrophages were fixed, and nuclei were stained with DAPI. Optical slices were visualized using fluorescence and phase contrast. Images represent the merged maximal intensity projections. Fluorescent bacteria harboring P*_mgtC_–gfp*[AAV] are indicated with white arrows. (**B**) Live imaging of infected macrophages was performed by epifluorescent microscopy, from 1.5 to 2 h post-infection. Brightfield and GFP channels were merged (scale bar = 10 µm).

We previously reported that expression of the *P. aeruginosa mgtC* gene is induced when the bacteria reside inside macrophages based on RT-qPCR analyses (~30-fold compared to 1 mM Mg^2+^) (11). Here, the reporter system was used to visualize *P. aeruginosa mgtC* promoter activation inside cultured macrophages. J774 macrophages were infected with strains expressing unstable GFP at a multiplicity of infection (MOI) of 10. After 25 min to enable phagocytosis, several washes were performed to remove adherent bacteria, and gentamicin was added to kill extracellular bacteria. Infected macrophages were fixed at 2 h post-phagocytosis, and structured illumination microscopy stacks were obtained using a fluorescence microscope. As anticipated, fluorescent bacteria were observed in cells infected with the strain harboring the constitutive *gyrA* promoter construct (P*_gyrA_–gfp*[AAV]), but not in those infected with the negative control (P_less_*–gfp*[AAV]) (Fig. 2A). With the strain harboring the *mgtC* promoter construct, bacteria with strong fluorescent signals were observed within macrophages (Fig. 2A), although in substantially lower numbers than with the positive control P*_gyrA_–gfp*[AAV]. This difference was not due to distinct phagocytic uptake rates (Fig. S2 available at https://doi.org/10.5281/zenodo.20323693), suggesting a heterogeneous bacterial population with only a limited subset having an activated *mgtC* promoter and/or an expression below the fluorescence detection threshold. Live imaging of macrophages infected with P*_mgtC_–gfp*[AAV] bacteria supported the notion of a transient intracellular expression of the *mgtC* promoter, as shown by the decrease in fluorescence over time (it is also possible that some fluorescent bacteria moved out of the optical slice over time) (Fig. 2B). The visualization of *mgtC* promoter activation inside macrophages, together with Fig. S1 available at https://doi.org/10.5281/zenodo.20323693 that showed a lack of expression in non-inducing conditions, is consistent with our previous RT-qPCR analysis (11).

**Fig 3:**
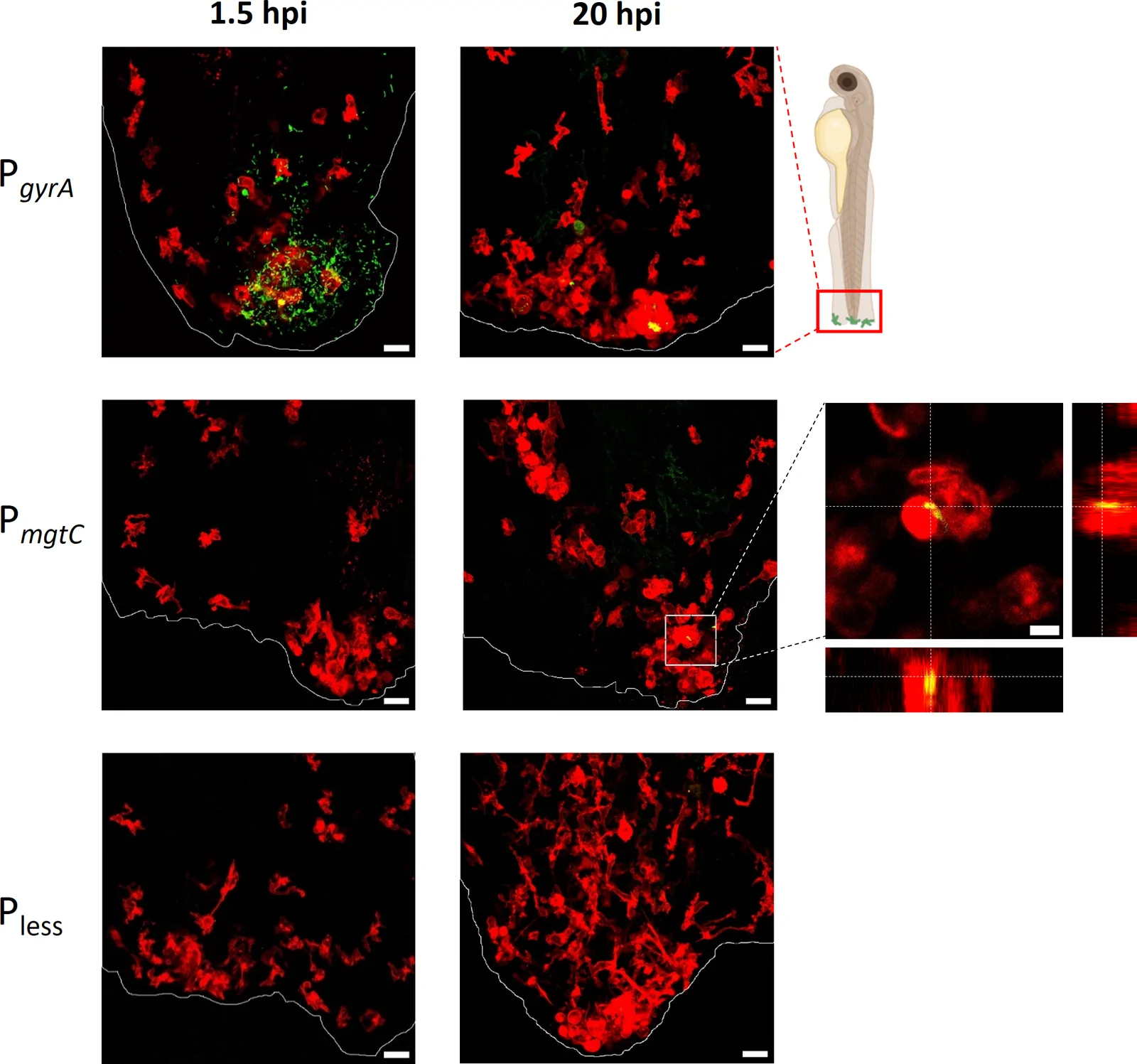
Live imaging of zebrafish larvae infected with PAO1 strains harboring *gfp*[AAV] reporters. *P. aeruginosa* PAO1 strain carrying the reporter gene *gfp*[AAV] under *gyrA* promoter (P*_gyrA_*), *mgtC* promoter (P*_mgtC_*), or without promoter (P_less_) was used to infect *tg(mfap4:mCherry-F)* zebrafish larvae after tail injury. Imaging was performed using a spinning-disk confocal microscope at 1.5 hpi or 20 hpi. Maximal intensity projections of larvae tails (tail limit indicated with a white line) are shown (scale bar = 20 µm). For P*_mgtC_* condition at 20 hpi, activation of *mgtC* promoter can be visualized, and an inset representing one optical section (scale bar = 5 µm) and associated orthogonal view is shown. Images were processed using Fiji, as described in the Materials and Methods section, except for the P*_gyrA_* condition at 1.5 hpi, where the maximum intensity display of the GFP channel was increased for better visualization of individual bacteria.

**Fig 4:**
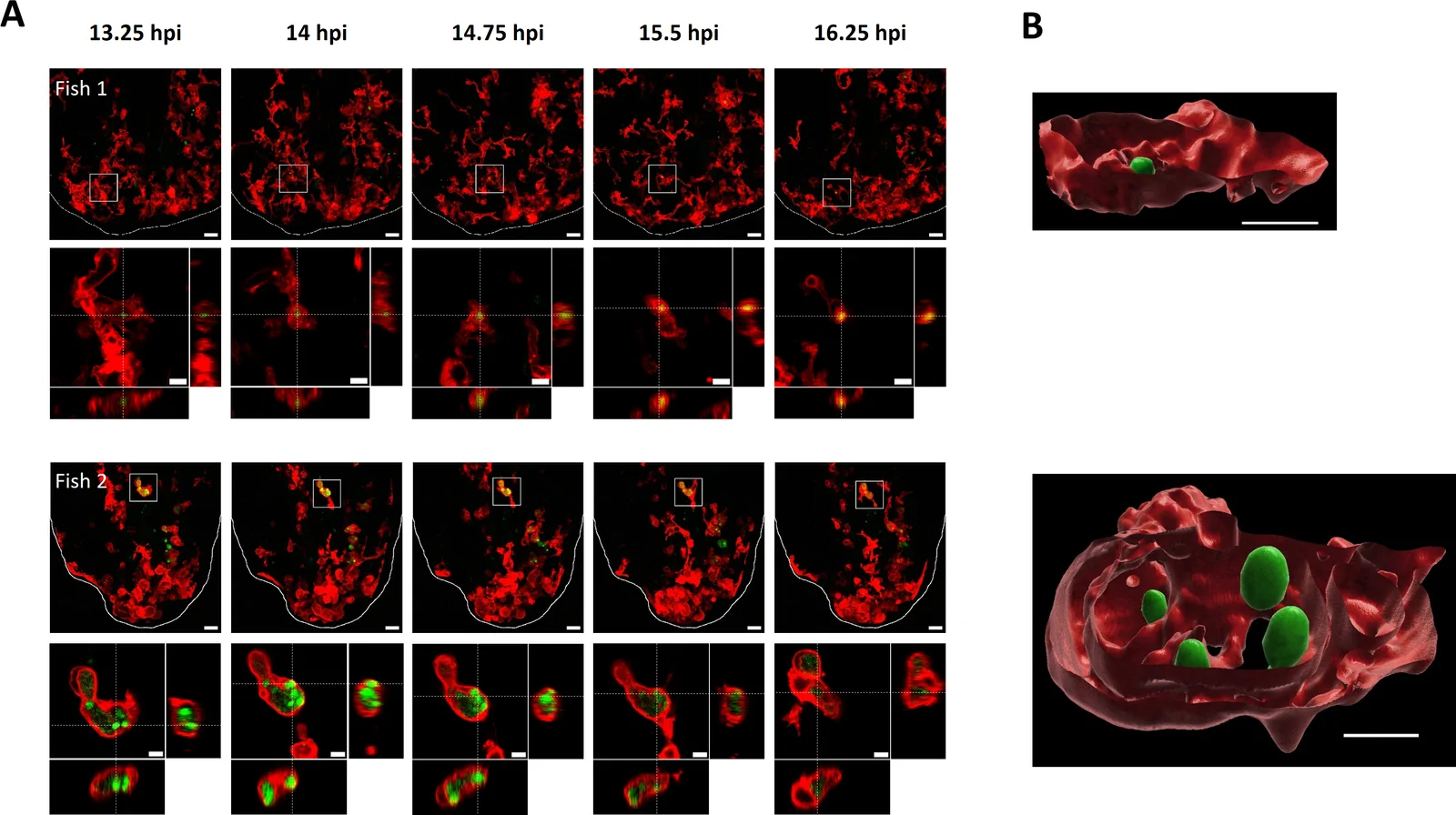
Time-lapse imaging to visualize *P. aeruginosa mgtC* promoter induction in macrophages of infected zebrafish larvae. *P. aeruginosa* PAO1 strain carrying the P*_mgtC_–gfp*[AAV] reporter construct was used to infect *tg(mfap4:mCherry-F)* zebrafish larvae after tail injury. Time-lapse imaging (every 45 min) was performed using a spinning-disk confocal microscope, and the time frame 13–16 hpi for two larvae is shown. (**A**) Maximal intensity projections of larvae tails (tail limit indicated with a white line) from two different larvae are shown (scale bar = 20 µm), as well as an inset representing one optical section (scale bar = 5 µm) and associated orthogonal views. (**B**) 3D reconstruction of the 13.25 hpi inset from both larvae, depicting one (fish 1) or four (fish 2) intramacrophagic green fluorescent events (scale bar = 5 µm).

We next investigated, for the first time, the activation of the *P. aeruginosa mgtC* promoter *in vivo*, using zebrafish (*Danio rerio*) larvae as an infection model. The zebrafish embryo is a unique vertebrate model to study macrophage-microbe interactions, thanks to its transparency, which allows visualization of both fluorescent macrophages, using specific transgenic fish lines, and fluorescent bacteria (26). Infection of zebrafish larvae previously enabled real-time visualization of *P. aeruginosa* phagocytosed by macrophages (6, 8–10). To assess *mgtC* promoter activity *in vivo*, we infected zebrafish larvae harboring red-fluorescent macrophages (*tg(mfap4:mCherry-F)*) with the reporter PAO1 strain carrying P*_mgtC_–gfp*[AAV], P*_gyrA_–gfp*[AAV], or P_less_*–gfp*[AAV]. We used an infection protocol based on immersion of tail-injured larvae in bacterial culture (10). Using this protocol, we previously showed an important reduction of PAO1 bacterial load in the first 24 h, which varies between individual larvae, and few persistent bacteria at 20 h post-infection (hpi) that can be found inside macrophages (10). Here, live confocal imaging was first performed 1.5 hpi or 20 hpi, and maximal projection images are shown in Fig. 3. As expected, at an early time after infection (1.5 hpi), bacterial colonization was clearly observed with the strain harboring the constitutively active *gyrA* promoter, and at 20 hpi, the number of fluorescent P*_gyrA_–gfp*[AAV] bacteria was much lower than that at 1.5 hpi. In contrast to the positive control, no fluorescent signal was detected with P*_mgtC_–gfp*[AAV] at an early time after infection (1.5 hpi). This is linked to a lack of GFP expression and not due to a defective infection, since bacterial colonization is similar for strains harboring P*_gyrA_–gfp*[AAV] or P*_mgtC_–gfp*[AAV], as shown by the quantification of colony-forming units (CFUs) at 1.5 hpi (Fig. S3 available at https://doi.org/10.5281/zenodo.20323693), and the percentage of larvae mortality is similar upon infection (around 40–50% at 20 h post-infection). Interestingly, at 24 hpi, very rare green fluorescent bacteria were detected with P*_mgtC_–gfp*[AAV], indicating an *in vivo* activation of the *mgtC* promoter. Moreover, orthogonal views from an individual optical slice showed an intramacrophage localization (Fig. 3). As expected, no fluorescent bacteria are found with the negative control P_less_*–gfp*[AAV] at both time points, despite efficient bacterial colonization at 1.5 hpi (Fig. S3 available at https://doi.org/10.5281/zenodo.20323693). To extend the analysis of the strain harboring P*_mgtC_–gfp*[AAV], time-lapse imaging was performed on live infected larvae at intermediate time points (5 to 18.5 hpi). Few fluorescent bacteria visible at several consecutive time points were found mainly on images acquired between 13 and 16 hpi, as shown on maximal intensity projections of two independent larvae (Fig. 4A). Examination of individual optical slices and orthogonal views (Fig. 4A), as well as 3D-reconstruction (Fig. 4B), confirmed the intramacrophage localization of some fluorescent bacteria. However, these fluorescence events remained rare, supporting the hypothesis of transient expression above the detection threshold under these acquisition conditions. Imaging of larvae infected with P*_mgtC_–gfp*[AAV] strain at 16 hpi was also conducted with increased laser power and exposure time, which provided a slightly brighter green signal and allowed the visualization of a few additional fluorescent bacteria (Fig. S4 available at https://doi.org/10.5281/zenodo.20323693). However, the settings were maintained at lower laser power/exposure time for all conditions to allow comparisons and avoid phototoxicity. Although rare under our experimental conditions, these experiments provide the first qualitative evidence that the *P. aeruginosa mgtC* promoter is not initially expressed but can be induced *in vivo*, and that *mgtC*-expressing bacteria can be visualized inside macrophages within a certain time frame. The rarity of *mgtC*-expressing bacteria is likely due to a combination of factors, including elimination of the PAO1 strain over time in the larvae, limitations in fluorescent signal detection, and, as observed *in vitro* and in cultured macrophages, *mgtC* expression in only a subset of bacteria.

### *P. aeruginosa* MgtC is regulated by the PhoP regulator but not its cognate sensor PhoQ

**Fig 5:**
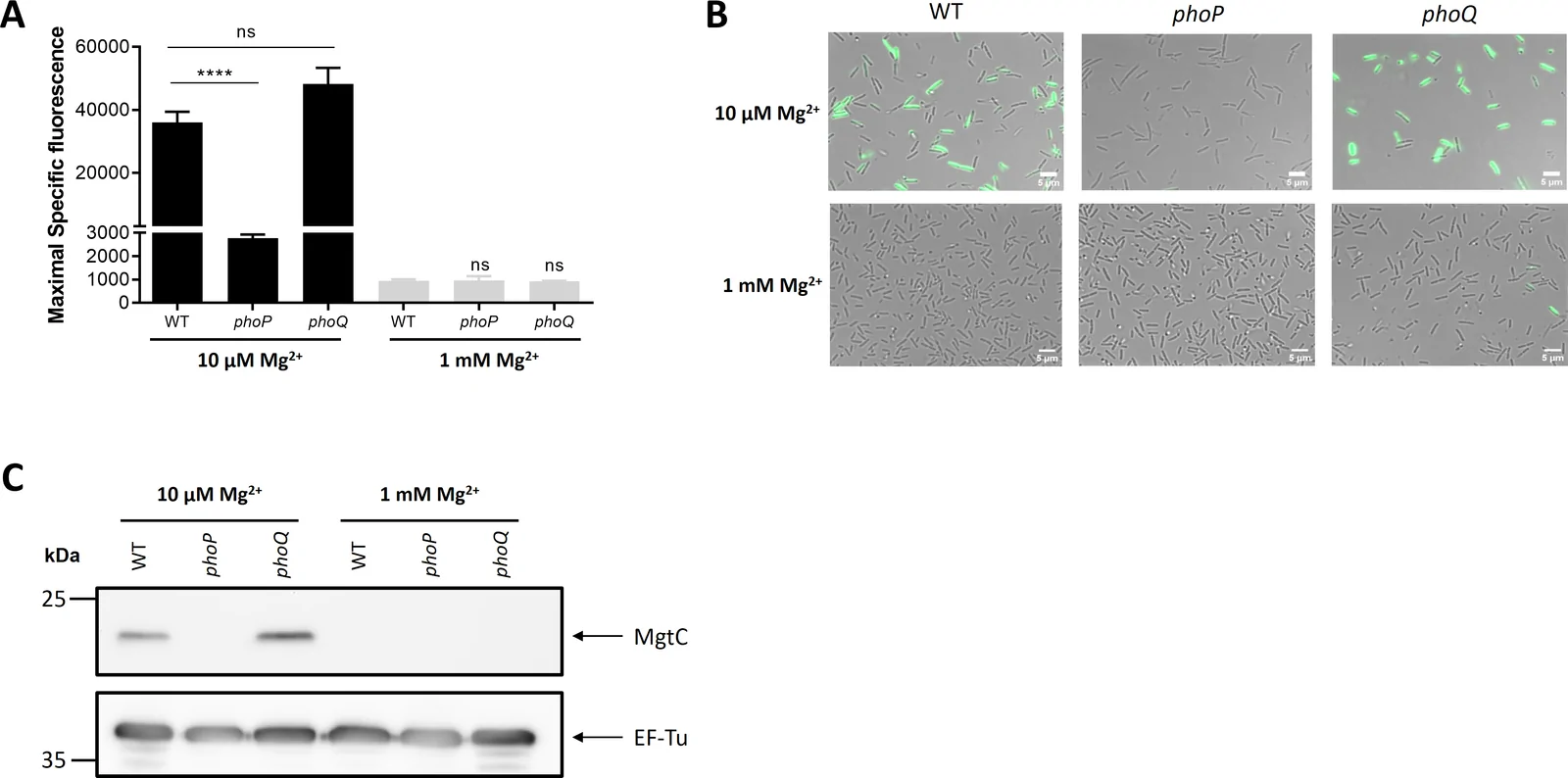
Magnesium-dependent activation of the *P. aeruginosa mgtC* promoter requires PhoP but not PhoQ. (**A**) Wild-type, *phoP*, and *phoQ* mutant strains harboring the P*_mgtC_–gfp*[AAV] plasmid were grown in NCE medium containing either 10 µM or 1 mM Mg^2+^. Specific fluorescence was measured and expressed as the ratio of GFP fluorescence to absorbance at 600 nm. The maximal specific fluorescence is shown. Data represent the mean ± standard error from three to six independent experiments. Statistical analysis (WT vs *phoP* or *phoQ*) was performed using an unpaired *t*-test (****P < 0.0001; ns, not significant). (**B**) Visualization of bacteria by fluorescence microscopy. Bacteria were grown for 2 h in NCE medium with 10 µM or 1 mM Mg^2+^. Merged phase-contrast and epifluorescence images are shown. (**C**) Assessment of MgtC protein levels by western blotting. Total lysates were prepared from cultures of PAO1 wild-type, *phoP*, and *phoQ* mutant strains incubated for 5 h in NCE medium with 10 µM or 1 mM Mg^2+^. Membranes were probed with MgtC and EF-Tu antibodies, the latter used as a loading control. A representative experiment from two independent replicates is shown.

Since *P. aeruginosa mgtC* is induced by Mg^2+^ depletion and inside macrophages, conditions also known to induce *mgtC* in *S.* Typhimurium, we hypothesized that *P. aeruginosa mgtC* might be regulated by the PhoPQ two-component regulatory system, as observed in *S.* Typhimurium (15, 27). To address this, we introduced the plasmid harboring the P*_mgtC_–gfp*[AAV] fusion into PAO1 *phoP* and *phoQ* mutant strains. Fluorescence quantification of strains grown in 10 µM Mg^2+^ medium showed a lack of *mgtC* promoter induction in the *phoP* mutant background, whereas induction was maintained in the *phoQ* mutant (Fig. 5A). Fluorescence microscopy provided consistent results, showing an absence of *mgtC* promoter induction in the *phoP* mutant and preserved induction in the *phoQ* mutant under 10 µM Mg^2+^ (Fig. 5B).

To further confirm that induction of *P. aeruginosa mgtC* was dependent on PhoP, but not on PhoQ, we assessed MgtC protein levels in wild-type, *phoP*, and *phoQ* mutant strains in both PAO1 and PA14 genetic backgrounds. Total lysates were prepared from bacteria grown in 10 µM or 1 mM Mg^2+^, and western blotting was carried out with anti-MgtC antibodies (28). As expected, MgtC was not expressed in 1 mM Mg,^2+^ but was induced in 10 µM Mg^2+^, both in the PAO1 (Fig. 5C) and PA14 (Fig. S5 available at https://doi.org/10.5281/zenodo.20323693) strains. Expression of MgtC in 10 µM Mg^2+^ was strongly reduced in the *phoP* mutant, but not in the *phoQ* mutant, in both genetic backgrounds (Fig. 5C; Fig. S5 available at https://doi.org/10.5281/zenodo.20323693). The complemented *phoP* mutant exhibited wild-type levels of MgtC expression (Fig. S6 available at https://doi.org/10.5281/zenodo.20323693). Taken together, these results indicate that magnesium-dependent regulation of MgtC is positively regulated by PhoP, but not the PhoQ sensor.

### *P. aeruginosa* MgtC is regulated by CbrAB

We hypothesized that MgtC might be regulated by another sensor kinase. Mutant strains for *P. aeruginosa* sensor kinases are available from a PA14 transposon mutant library and have been phenotypically characterized (29). Based on their characteristics, we selected and tested 22 kinases as potential regulators of MgtC. First, MgtC protein expression was assessed in these kinase mutant strains grown in 10 µM Mg^2+^ and compared with wild-type PA14 (Fig. S7 available at https://doi.org/10.5281/zenodo.20323693). A defect in MgtC induction was observed only in the *cbrA* transposon mutant (Fig. S7 available at https://doi.org/10.5281/zenodo.20323693), and a similar finding was observed using a *cbrA* deletion mutant (Fig. S8A available at https://doi.org/10.5281/zenodo.20323693). We then tested a mutant lacking the cognate response regulator *cbrB* and similarly observed a defect in MgtC induction under magnesium-deprived conditions (Fig. S8A available at https://doi.org/10.5281/zenodo.20323693). Complemented *cbrA* and *cbrB* mutants had a wild-type level of MgtC expression (Fig. S8B available at https://doi.org/10.5281/zenodo.20323693).

**Fig 6:**
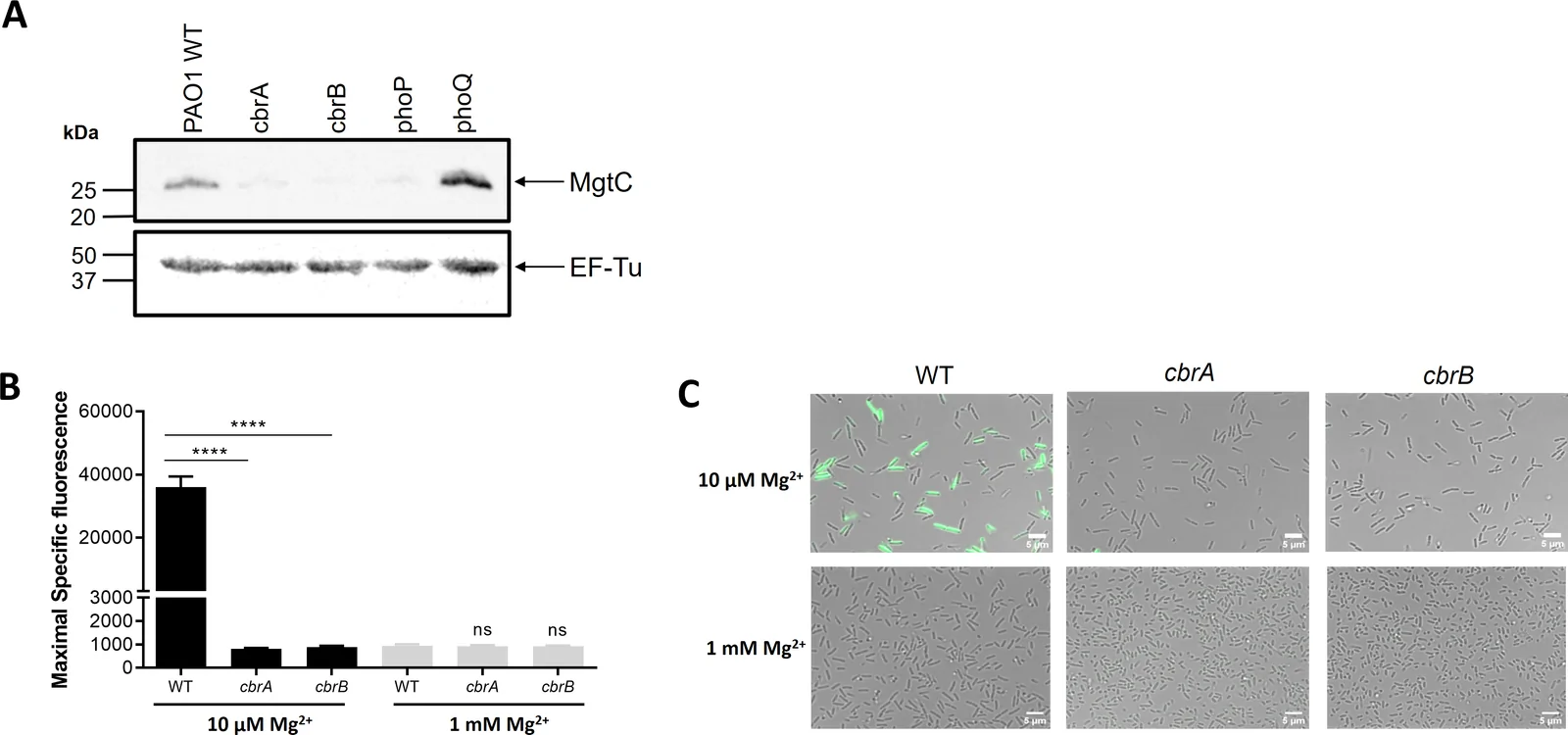
Magnesium-dependent regulation of *P. aeruginosa* MgtC expression involves CbrA and CbrB. (**A**) Western blot analysis of MgtC protein levels. Total lysates were prepared from cultures of various strains (PAO1 background) incubated for 5 h in NCE medium with 10 µM Mg^2+^. Membranes were probed with MgtC and EF-Tu antibodies, the latter serving as a loading control. A representative experiment from two independent replicates is shown. (**B**) Wild-type, *cbrA*, and *cbrB* mutant strains harboring the P*_mgtC_–gfp*[AAV] plasmid were grown in media containing in NCE medium with 10 µM or 1 mM Mg^2+^. Specific fluorescence was measured and expressed as the ratio of GFP fluorescence to absorbance at 600 nm. The maximal specific fluorescence intensity is reported. Data represent the mean ± standard error from three independent experiments. Statistical analysis (WT vs *cbrA* or *cbrB*) was performed using an unpaired *t*-test (****P < 0.0001; ns, not significant). (**C**) Fluorescence microscopy visualization of bacteria grown for 2 h in NCE medium with 10 µM or 1 mM Mg^2+^. Merged phase-contrast and epifluorescence images are shown. The images for wild-type strain are duplicates of Fig. 5B.

Regulation of MgtC expression by CbrA and CbrB was further confirmed in the PAO1 background by western blotting (Fig. 6A). To quantify this effect in low magnesium conditions, the plasmid harboring the P*_mgtC_–gfp*[AAV] fusion was introduced into *cbrA* and *cbrB* mutants in the PAO1 background. Fluorescence quantification (Fig. 6B), together with fluorescence microscopy (Fig. 6C), confirmed that *mgtC* promoter induction in 10 µM Mg^2+^ was impaired in both *cbrA* and *cbrB* mutants.

These findings support a model in which the *P. aeruginosa mgtC* promoter is regulated not only by PhoP, but also by the CbrAB two-component system. Although our results suggest that PhoP activation may involve a cross-talk with another histidine kinase, such as CbrA, demonstrating a direct phosphorylation event remains technically challenging. The phosphoryl group on the conserved aspartate of response regulators is intrinsically unstable, which complicates biochemical detection of PhoP phosphorylation *in vitro. In vivo*, additional layers of complexity arise from the potential contribution of multiple kinases or small-molecule phosphodonors, making it difficult to unambiguously assign the source of PhoP phosphorylation.

### PhoP and CbrB can bind to the *P. aeruginosa mgtC* promoter

**Fig 7:**
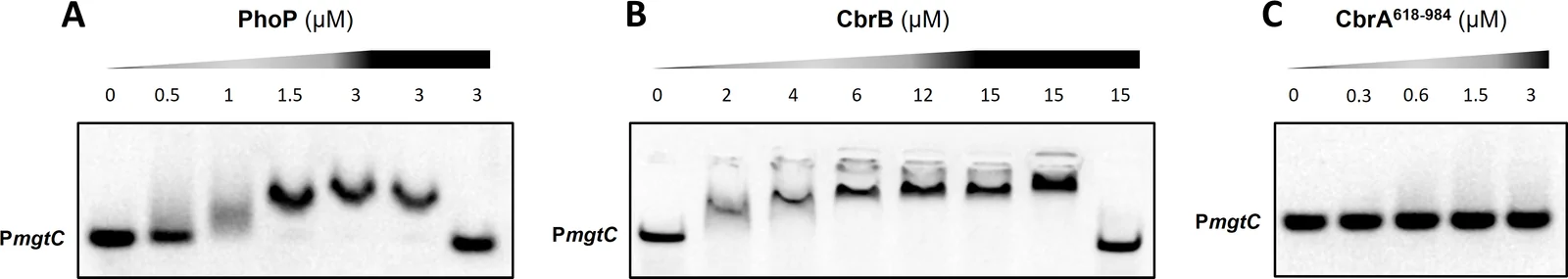
PhoP and CbrB bind to the *P. aeruginosa mgtC* promoter region. Electrophoretic mobility shift assays (EMSAs) were performed using purified recombinant N-terminally 6x-His-tagged PhoP (**A**), CbrB (**B**), or CbrA^618-984^ (**C**) and a DNA probe corresponding to the *mgtC* promoter region. The concentration of the His-tagged proteins is indicated. The shifted bands observed with PhoP and CbrB were not competed by a non-specific DNA fragment corresponding to an unrelated PCR DNA fragment (penultimate lane), but were competed by the corresponding unlabeled probes (last lane). The CbrA^618-984^ does not bind to the *mgtC* promoter region (negative control). All DNA probes were labeled with DY682. Data shown are representative of two independent experiments.

To test whether PhoP and CbrB directly interact with the *P. aeruginosa mgtC* promoter, the His-tagged PhoP and CbrB proteins were purified. Electrophoretic mobility shift assays (EMSA) were then performed to evaluate their ability to bind the *mgtC* promoter region. EMSA demonstrated that PhoP can directly bind the *mgtC* promoter region (Fig. 7A). A similar result was obtained with CbrB, although larger amounts of purified His-CbrB protein were required to shift the DNA fragment containing the *mgtC* promoter (Fig. 7B). The observed shifts were specific, as they could be out-competed by excess unlabeled *mgtC* promoter DNA, but not by an unrelated DNA fragment (Fig. 7A and B). As expected, a control protein (CbrA^618-984^) did not bind the *mgtC* promoter region (Fig. 7C). These data support the conclusion that both PhoP and CbrB directly regulate *mgtC* expression through binding to its promoter region.

## DISCUSSION

Bacterial genes involved in the physiological adaptation to the host intracellular microenvironment require tight and rapid regulation. In this study, we developed a reporter system to monitor in real time the activation of the *P. aeruginosa mgtC* promoter and visualized its activation inside macrophages, both in cultured cells and *in vivo* in a vertebrate model. Moreover, we showed that *P. aeruginosa mgtC* expression was regulated through a mechanism distinct from that of *Salmonella* MgtC, despite being induced by similar environmental signals.

The transcriptional fusion with the reporter gene *gfp*[AAV], encoding an unstable GFP, proved to be a powerful tool to evaluate *mgtC* promoter activity both *in vitro*, in macrophages, and *in vivo*, allowing dynamic single-cell analysis. Using this system, we detected activation of the *P. aeruginosa mgtC* promoter in low-magnesium medium and during intracellular survival in cultured macrophages. Unlike the constitutive *gyrA* promoter used as a control, *mgtC* promoter activation was observed only in a subset of bacteria *in vitro*, which can be due to a combination of features, including transient or heterogeneous expression. The effect appeared even more pronounced inside cultured macrophages, where expression may be also restricted to specialized subpopulations and/or subcellular compartments. Notably, we also visualized *mgtC* promoter induction *in vivo* using the zebrafish vertebrate model. Due to its transparency, the zebrafish embryo allows simultaneous visualization of fluorescent host cells and bacteria, and this is the first report of *P. aeruginosa* gene induction observed *in vivo* within macrophages. Time-lapse analysis suggested a window of expression around 13–16 hpi under our infection conditions, but further analyses will be required to strengthen this observation. However, fluorescent bacteria remained rare, probably due to a combination of factors, including transient activation, heterogeneity, specialized subpopulations, unstable fluorophore, and clearance of PAO1 bacteria over time. Further studies are required to better characterize the subset of bacteria expressing *mgtC in vivo*, and the methodology has to be optimized :using a *P. aeruginosa* strain known to persist in zebrafish larvae (10) to overcome bacterial clearance, a dual reporter system to provide an internal fluorescent control, and stable, bright fluorophores to mitigate detection limitations.

To investigate the regulatory mechanism of *P. aeruginosa* MgtC, we focused on a two-component system involved in magnesium-responsive gene activation, which allows rapid adaptation to changing environments. In *Salmonella*, *mgtC* regulation is mediated by the PhoP regulator and its cognate sensor PhoQ (27). In *P. aeruginosa*, previous microarray analysis did not identify *mgtC* as a PhoP-regulated gene (20), possibly due to technical limitations linked to the inferior sensitivity of microarrays and/or the use of an insufficiently stringent magnesium condition (10 µM here versus 20 µM in the previous study). Using *phoP* mutant strains, we demonstrated here that the *P. aeruginosa mgtC* promoter is indeed regulated by PhoP, and accordingly, we observed direct binding of PhoP to the *mgtC* promoter in EMSA assays. In contrast, induction was maintained in a *phoQ* mutant and was thus independent of PhoQ. This supports previous results showing that PhoQ proteins from *P. aeruginosa* and *Salmonella* differ structurally and functionally (21), with *P. aeruginosa* PhoQ acting primarily as a phosphatase that dephosphorylates the cognate regulatory protein PhoP (30) rather than as a kinase (23). Thus, *phoQ* inactivation leads to increased PhoP phosphorylation and constitutive activation of its targets, which is consistent with the higher *mgtC* promoter activity observed in the *phoQ* mutant. This is also in line with previous transcriptomic data reporting a ~3-fold upregulation of *mgtC* in a *phoQ* mutant (22).

Since the sensor kinase responsible for PhoP phosphorylation in *P. aeruginosa* remains unknown, we explored alternative regulatory pathways. We identified CbrA as a key kinase involved in *mgtC* regulation. Interestingly, its cognate response regulator, CbrB, is also involved and directly binds to the *mgtC* promoter, although with lower affinity than PhoP. The precise contributions of each regulator to the fine-tuned control of *mgtC* promoter activation, however, remain to be fully elucidated. CbrAB is a two-component system specific to *Pseudomonas* species, involved in adaptive responses to nutritional changes and *P. aeruginosa* virulence (31). Carbon sources and carbon catabolite repression modulate this two-component system, and its activating signal, although not identified, could be related to the C:N balance (32). CbrA is not a canonical histidine kinase as it is a fusion protein composed of a membrane-integrated transporter followed by the autokinase core domain. The implication of CbrA in virulence in a mouse infection model independently of CbrB supports the hypothesis of a crosstalk between CbrA and another regulatory system (33). Interestingly, a potential cross-talk between CbrA and PhoP has been previously suggested based on common phenotypes between *cbrA* and *phoP* mutants, such as increased sensitivity to polymyxin B (34). Moreover, *cbrA* and *mgtC* mutants also share common phenotypes, such as hyper-biofilm formation (11, 35) and increased phagocytosis (11, 33). In addition, both CbrA-CbrB and MgtC interplay with the PhoB regulon, which regulates phosphate transport (36–38). These shared traits are consistent with the hypothesis that some of the phenotypes observed in the *cbrA* mutant could be attributed, at least in part, to downstream regulation of the *mgtC* gene. In addition to cross-phosphorylation by an alternative histidine kinase such as CbrA, another possible mechanism for PhoP activation is phosphorylation by small-molecule phosphodonors such as acetyl phosphate. This metabolite has been reported to phosphorylate response regulators in other bacteria when the cognate sensor kinase is absent, thereby providing an alternative regulatory route (39). Such a mechanism has indeed been reported for a *Salmonella* PhoP mutant protein (40) and could therefore also contribute to *Pseudomonas* PhoP activation. How *Pseudomonas* MgtC is regulated by magnesium remains an open question, and two possible explanations are that CbrA might sense Mg^2+^ or that indirect signaling may occur through another molecule.

In conclusion, we developed a sensitive and versatile fluorescent reporter system that enables real-time visualization of *P. aeruginosa mgtC* promoter activation within macrophages, both in cultured cells and in a living host. This approach, combined with the use of the zebrafish embryo model, could be readily adapted to other pathogens to track bacterial gene expression *in vivo* and dissect host-cell specific activation patterns, complementing global dynamic approaches (41). Our findings also revealed that the MgtC virulence factor is regulated through both conserved and species-specific mechanisms in *P. aeruginosa* compared to classical intracellular bacteria, reflecting the distinct, yet overlapping, lifestyles of these pathogens.

## MATERIALS AND METHODS

### Bacterial strains and growth conditions

Bacterial strains and plasmids are described in Table 1. In addition, the following sensor kinase mutants of the PA14 Harvard transposon insertion library (42) were used: *fpvR* (ID33780), *parS* (ID41270), *czcS* (ID31950), *copS* (ID27800), *cprS* (ID24340), *pmrB* (ID63160), *amgS* (ID68680), *phoR* (ID70760), *pvrS* (ID59800), *wspE* (ID16470), *cbrA* (ID62530), *mifS* (ID72740), *fleS* (ID50200), *pirS* (ID52240), *mucR* (ID42220), *pfeS* (ID29360), *rocS2* (ID24720), *gtrS* (ID22960), *rocS1* (ID12820), *bfiS* (ID09680), *roxS* (ID58320), and *kinB* (ID72390). *P. aeruginosa* strains were grown at 37°C in Luria broth (LB). Growth in magnesium-defined medium was performed in No-carbon-E (NCE) minimal medium (43), supplemented with 0.1% casamino acids, 38 mM glycerol, and containing 10 µM or 1 mM MgCl_2_. Plasmids expressing unstable GFP were introduced in *P. aeruginosa* by conjugation, using an *E. coli* strain containing helper plasmid pRK2013. Recombinant bacteria were selected on LB agar plates containing 15 µg/mL triclosan and 500 μg/mL kanamycin.

**TABLE 1:** Strains and plasmids used in this study

Strain	Description	Reference or source
*P. aeruginosa*		
PAO1	Wild-type *P. aeruginosa*	Laboratory collection
H851	PAO1 *phoP*::*xylE-*Gm^R^	(23)
H854	PAO1 *phoQ*::*xylE-*Gm^R^	(23)
PAO1 *cbrA*	Transposon insertion (ID33836)	(34)
PAO1 *cbrB*	*∆cbrB*	(34)
PA14		Laboratory collection
PA14 *phoP*	Transposon insertion Gm^R^ (ID49180)	(42)
PA14 *phoQ*	Transposon insertion Gm^R^ (ID49170)	(42)
PA14 *cbrA*	*∆cbrA*	(34)
PA14 *cbrB*	Transposon insertion Gm^R^ (ID44074)	(42)
H851 compl	*phoP*::*xylE-*Gm^R^ + pEMPQ2 a (*phoPQ*^+^)	(23)
PA14 *cbrA* compl	*∆cbrA* + pUCP18::*cbrAB*^+^	(34)
PA14 *cbrB* compl	*cbrB*::Gm^R^ + pUCP18::*cbrAB*^+^	(34)
*E. coli*		
BL21(DE3)Star	*S*train allowing high-level recombinant protein expression. IPTG-inducible T7 RNA polymerase	Invitrogen
Plasmids		
pETPhos	pET15b (Novagen) derivative including the replacement of the thrombin site coding sequence with a tobacco etch virus protease site and Ser to Gly mutagenesis in the N-terminal His tag (AmpR)	(44)
pETPhos_CbrA^618-984^	pETPhos derivative used to express His-tagged fusion of *P. aeruginosa* CbrA^618-984^ in *E. coli*	This study
pETPhos_CbrB	pETPhos derivative used to express His-tagged fusion of *P. aeruginosa* CbrB in *E. coli*	This study
pETPhos_PhoP	pETPhos derivative used to express His-tagged fusion of *P. aeruginosa* PhoP in *E. coli*	This study
pRK2013	Tra^+^, Mob^+^, ColE1, KmR	Laboratory collection
pPROBE*gfp*[AAV]	Promoterless unstable GFP	(25)
P*_mgtC_–gfp*[AAV]	Promoter *mgtC* unstable GFP	This study
P*_gyrA_–gfp*[AAV]	Promoter *gyrA* unstable GFP	This study

### Construction of plasmids expressing *gfp*[AAV] under the control of the *mgtC* gene promoter or a constitutive promoter

We obtained a plasmid expressing the reporter gene *gfp*[AAV] under the control of the *mgtC* gene promoter by amplifying a DNA fragment (500 nucleotides) located upstream *mgtC* by colony PCR from PAO1 strain, using primers described in Table S1 available at https://doi.org/10.5281/zenodo.20323693. The PCR product was digested and inserted into the corresponding sites of pPROBE*–gfp*[AAV] vector, yielding P*_mgtC_–gfp*[AAV]. We chose the *gyrA* promoter as a constitutive control because the *gyrA* gene was previously identified as similarly expressed in 10 µM or 1 mM Mg^2+^ medium and inside macrophages (11). The plasmid P*_gyrA_–gfp*[AAV] was constructed by inserting the *gyrA* promoter region (500 nucleotides). A promoterless vector (P_less_*–gfp*[AAV]) is used as a negative control. Plasmids were first introduced into chemically competent E. cloni 10G (Lucigen) before conjugation into the PAO1 strain.

### Analysis of the expression of the *gfp*[AAV] constructs *in vitro*

Bacteria were grown overnight in LB medium supplemented with 200 μg/mL kanamycin. Bacteria were diluted 1:50 in LB and grown until OD_600nm_ of approximately 0.6–0.7. Bacteria were washed twice in NCE medium without Mg^2+^ and resuspended in NCE medium supplemented with 10 µM or 1 mM Mg^2+^. Bacterial cultures were transferred in triplicate into sterile black sided, clear-bottomed 96-well plates (Greiner) and incubated for 5–6 h at 37°C with orbital shaking in Tecan microplate reader (Infinite M200 for Fig. 1B or Spark 20M microplate reader for Fig. 5 and 6). Absorbance at 600 nm and GFP fluorescence intensity, with excitation at 485 nm and emission at 520 nm, were measured every 30 min. Specific fluorescence was obtained by dividing fluorescence units by the absorbance value. For each condition, the maximal value of specific fluorescence is reported on the graphs.

### Analysis of bacterial cultures by epifluorescence microscopy

Bacteria were grown overnight in LB medium supplemented with 200 μg/mL kanamycin. The cultures were then diluted 1:10 in fresh LB and grown until reaching an OD_600nm_ of approximately 0.7–0.8. Cells were washed twice in NCE medium either lacking Mg²^+^ or supplemented with 1 mM Mg²^+^ and subsequently diluted in the corresponding NCE medium. A 250 µL suspension was plated into a glass-bottom µ-slide (8-well, Ibidi 80827) previously coated with 250 µL of 50 µg/mL poly-D-lysine (Gibco A38904-01) 2 h at 37°C, followed by two phosphate-buffered saline (PBS) washes and one wash in H₂O. After centrifugation at 200 × *g* for 5 min, the medium was replaced with fresh NCE containing either 10 µM or 1 mM Mg²^+^. The specimen was inserted into an incubation chamber set at 37°C, and images were acquired using a Plan-Apochromat 63× (NA = 1.40) objective mounted on a Zeiss AxioObserver Z1/7 inverted microscope equipped with an Axiocam 503 CCD camera. Brightfield and FITC channels were used. Images were processed using Fiji (https://imagej.net/software/fiji/): brightfield displays were adjusted to ensure consistent contrast between images, while GFP display settings were kept identical across all conditions.

### Fluorescence imaging of infected J774 macrophages

J774 cells (murine macrophage cell line J774A.1) were maintained at 37°C in 5% CO_2_ in Dulbecco’s modified Eagle medium (DMEM) (Gibco 41965) supplemented with 10% heat-inactivated fetal bovine serum (FBS) (Gibco 10270106). J774 macrophages were seeded on glass coverslips in 24-well plates, and infection by GFP-expressing *P. aeruginosa* was carried out essentially as described previously (11). Mid-log phase *P. aeruginosa* grown in LB broth was centrifuged, washed in PBS, and resuspended in complete DMEM to infect J774 macrophages (5 × 10^5^ cells/well) at an MOI of 10. After centrifugation of the 24-well culture plate for 5 min at 200 × *g* for synchronization of infection, bacterial phagocytosis was allowed for 25 min. Cells were washed three times with sterile PBS, and fresh complete DMEM medium supplemented with 400 μg/mL gentamicin was added.

After 2 h, cells were fixed with 4% paraformaldehyde (EMS, USA) in PBS for 30 min, washed once with PBS, and coverslips were mounted on glass slides in Vectashield with DAPI (Vector Laboratories, Inc.). The slides were examined using an upright fluorescence microscope (Axioimager Z1, Zeiss) equipped with an Apotome 1 for optical sectioning. A 63× Apochromat objective (NA 1.4) was used, and transmitted light was acquired using differential interference contrast (DIC). FITC filter was used to visualize GFP-expressing bacteria. Cell nuclei were visualized using DAPI filter. Z-stacks were systematically acquired using a Z-step of 240 nm. Images were processed using ZEN software (Zeiss).

Alternatively, the dynamics of *mgtC* promoter activation were assessed using time-lapse microscopy. One day before infection, J774 cells were seeded in glass-bottom µ-slide (8-well, Ibidi 80827) at a density of 1.25 × 10^5^ cells/cm^2^. Bacteria were grown and prepared as described earlier to perform infection at an MOI of 10. Centrifugation, phagocytosis, and washes were the same as before. Finally, infected cells were incubated in complete DMEM lacking phenol red and containing 400 µg/mL gentamicin. Cells were imaged using an inverted epifluorescence microscope (AxioObserver.Z1, Zeiss), equipped with an incubation chamber set at 37°C and 5% CO_2_, and an AxioCam 503 Mono CCD camera. Time-lapse experiments (one image each 10 min) were performed by automatic acquisition of random fields using a 63× plan-apochromat objective (NA 1.4). Fiji was used for image processing.

### Infection of *Danio rerio* larvae by immersion

A fish line carrying red-fluorescent macrophages, Tg(*mfap4:mCherry-F*)ump6Tg (45), was used. Fish maintenance, staging, and husbandry were performed at 28°C as described (46). Bacterial strains that were freshly streaked out from glycerol stocks were grown in LB medium to mid-log phase (OD_600nm_ = 0.8), recovered by centrifugation, and resuspended in fish water. Larvae at 2 days post-fertilization (dpf) were dechorionated, anesthetized with 0.02% tricaine, and tail injury was performed immediately before balneation in bacterial suspension, as described previously (10, 47).

### Live imaging of infected zebrafish larvae

For live imaging, anesthetized infected larvae were mounted in 35 mm glass bottom dishes (Ibidi 81218) and immobilized with 1% low melting-point agarose. DIC and fluorescence imaging were performed using a 40× (NA = 1.15, long working distance) water objective mounted on a Nikon Ti Eclipse inverted microscope coupled to a Yokogawa CSU-W1 spinning disk confocal system and an Andor Neo 5.5 sCMOS camera; focal planes covering the whole thickness of larvae caudal fin were acquired in steps of 1 µm. Time-lapse recordings were performed from 5 to 18.5 hpi, every 45 min at 28°C. Images were processed using Fiji, with the minimum and maximum intensity display of each channel kept equal between images. For visualization of bacteria localization related to macrophages, the Surfaces tool in Imaris software was used to reconstruct the 3D surfaces of both macrophages and bacteria.

### Bacterial load measurement in infected larvae

Before CFU quantification, infected larvae were washed for a few minutes in 4 mL of fish water and subsequently crushed individually using a pestle in 100 µL of PBS. Then, 100 µL of Triton X-100 (1% final concentration) was added for 10 min to liberate bacteria from residual cells/tissues. Following lysis, serial dilutions in PBS were spotted on LB agar plates and incubated approximately 18 h at 37°C before colony counting.

For larvae that were imaged and subsequently quantified for their bacterial burden, individuals were first immobilized in 1% low-melting agarose and imaged by confocal microscopy as described above. After imaging, larvae were released from the agarose for bacterial burden quantification.

### Preparation of lysates from bacterial cultures

Overnight culture of *P. aeruginosa* grown in LB medium supplemented with 50 µM MgCl_2_ was diluted 1:5 in LB and grown until OD_600nm_ of 0.8 to 1. Bacteria were washed in NCE medium without magnesium and were resuspended in the same medium supplemented with 10 µM Mg^2+^ or 1 mM Mg^2+^. Bacteria were harvested after 4–5 h of incubation at 37°C. To prepare whole-cell extracts, cultures were normalized to OD_600nm_, centrifuged, resuspended in Laemmli buffer, and lysed by heating at 95°C for 5 min before performing western blot assay.

### Western blot analysis

Total bacterial lysates were electrophoresed on 12.5% SDS-PAGE gels and transferred onto nitrocellulose membrane (Invitrogen) for immunoblotting. Rabbit anti-PA4635 (28) was used at 1:2,000 dilution, and mouse anti-EF-Tu antibody (48) was used at 1:20,000 dilution. Anti-rabbit or anti-mouse antibodies (dilution 1:30,000), labeled with fluorescent dye DyLight 800 (Thermo Scientific), were used for the detection of PA4635 and EF-Tu using LICOR Odyssey Fc Imaging System (excitation 783 nm and emission 797 nm) to quantify the amount of proteins. In Fig. 5C; Fig. S1A, S6 and S8B available at https://doi.org/10.5281/zenodo.20323693, 13.5% SDS-PAGE was used, and transfer was performed onto immobilon-P PVDF membrane (Merck IPVH00010), followed by incubation with HRP-conjugated secondary antibodies (Jackson ImmunoResearch, dilution 1:10,000) before signal detection in a Bio-Rad ChemiDoc imaging system and enhanced chemiluminescence substrates (Bio-Rad Clarity Max).

To assess the respective stability of MgtC and GFP[AAV] proteins, an overnight culture (LB medium with 200 μg/mL kanamycin) of the PAO1 strain harboring P*_mgtC_–gfp*[AAV] was diluted 1:10 in LB and grown until an OD_600nm_ of approximately 0.7. Bacteria were washed in NCE medium without Mg^2+^ and diluted 1:5 in NCE medium supplemented with 10 µM Mg^2+^. After 3 h of growth in this inducing condition, a shift to high magnesium (1 mM Mg^2+^) was performed to stop activation of the *mgtC* promoter. Bacterial lysates were prepared at different time points and normalized according to OD_600nm_. MgtC level was determined as described above using PVDF membrane, and GFP[AAV] level was determined using mouse monoclonal anti-GFP (Roche 11814460001) at 1:2000 dilution.

### Cloning, expression, and purification of recombinant CbrA^618-984^**, CbrB, and PhoP proteins**

The plasmids were constructed using NEB Gibson Assembly kit (New England Biolabs) and TOP10 competent bacteria (Invitrogen). The *cbrA*^618-984^, corresponding to the soluble CbrA domain, and *cbrB* and *phoP* gene fragments were amplified by PCR using *P. aeruginosa* PAO1 chromosomal DNA as a template with the primers listed in Table S1 available at https://doi.org/10.5281/zenodo.20323693. PCR fragments were Gibson cloned into the NdeI/BamHI-digested pETPhos vector (44), generating pETPhos_CbrA^618-984^, pETPhos_CbrB, and pETPhos_PhoP derivatives plasmids, respectively (Table 1). *E. coli* BL21 Star cells transformed with pETPhos_CbrA^618-984^ or pETPhos_PhoP were grown in LB (LB Broth MILLER, #1.10285.0500 Merck Millipore) medium containing 1 mg/mL of glucose and 100 µg/mL of ampicillin. CbrA^618-984^-expressing bacteria were grown at 37°C until OD_600_ reached 0.8 and induced with 0.5 mM IPTG overnight at 20°C. PhoP-expressing bacteria were grown at 37°C until an OD_600_ of 0.8 and induced with 0.5 mM IPTG overnight at 25°C. *E. coli* BL21 Star cells transformed with pETPhos_CbrB were grown at 37°C in TB (Terrific Broth Medium, #X972.1 Carl Roth) medium containing 1 mg/mL of glucose and 100 µg/mL of ampicillin until an OD_600_ of 0.3, followed by incubation at 30°C until an OD_600_ of 0.5, and induced with 0.5 mM IPTG overnight at 16°C. Bacterial cells were disrupted in a French pressure cell and centrifuged at 14,000 rpm for 25 min. Purifications were performed using TALON metal affinity resin (Takara, #635501) according to manufacturer’s instructions and eluted in 200 mM imidazole, 50 mM Tris-HCl (pH 7.4), 300 mM NaCl, and 10% (vol/vol) glycerol.

### Electrophoretic mobility shift assays

To assess DNA binding of CbrB and PhoP, the promoter region for *mgtC* (500 bp) was amplified using DYE682-labeled primers (Eurofins Genomics) (Table S1 available at https://doi.org/10.5281/zenodo.20323693), and the PCR products were purified using a QIAquick PCR purification kit (Qiagen). Indicated amounts of purified His-CbrB and His-PhoP were mixed with no more than 100 fmoles of DNA in EMSA buffer (20 mM HEPES [pH 8.0], 10 mM KCl, 2 mM MgCl_2_, 0.1 mM EDTA, 0.1 mM dithiothreitol, 50 μg/mL bovine serum albumin, and 10% glycerol) in a final volume reaction of 20 μL. After incubation at room temperature for 30 min, the reactions were run on a 2.5% agarose gel in cold TAE (Tris-acetate-EDTA) buffer (49). The gel was then imaged using the 700 nm channel of the Odyssey Infrared Imaging System (LI-COR).

### Statistical analysis

GraphPad Prism 8.3.0 was used to perform all statistical analyses and generate graphs. The statistical test applied to each data set was selected based on the normality of the data. The specific tests used for each analysis are indicated in the legends of the corresponding figures.
